# Metabolic and Pharmacokinetic Differentiation of STX209 and Racemic Baclofen in Humans

**DOI:** 10.3390/metabo2030596

**Published:** 2012-09-11

**Authors:** Raymundo Sanchez-Ponce, Li-Quan Wang, Wei Lu, Jana von Hehn, Maryann Cherubini, Roger Rush

**Affiliations:** 1 Seaside Therapeutics, Inc., 840 Memorial Drive, Cambridge, MA 02139, USA; Email: jvonhehn@seasidetherapeutics.com (J.H.); mcherubini@seasidetherapeutics.com (M.C.); rrush@seasidetherapeutics.com (R.R.); 2 XenoBiotic Laboratories, Inc., 107 Morgan Lane, Plainsboro, NJ 08536, USA; Email: LiQuan_Wang@xbl.com (L.-Q.W.) and wei_lu@xbl.com (W.L.)

**Keywords:** pharmacokinetics, baclofen, R-baclofen, metabolic differentiation

## Abstract

STX209 is an exploratory drug comprising the single, active R-enantiomer of baclofen which is in later stage clinical trials for the treatment of fragile x syndrome (FXS) and autism spectrum disorders (ASD). New clinical data in this article on the metabolism and pharmacokinetics of the R- and S-enantiomers of baclofen presents scientific evidence for stereoselective metabolism of only S-baclofen to an abundant oxidative deamination metabolite that is sterically resolved as the S-enantiomeric configuration. This metabolite undergoes some further metabolism by glucuronide conjugation. Consequences of this metabolic difference are a lower C_max_ and lower early plasma exposure of S-baclofen compared to R-baclofen and marginally lower urinary excretion of S-baclofen after racemic baclofen administration. These differences introduce compound-related exposure variances in humans in which subjects dosed with racemic baclofen are exposed to a prominent metabolite of baclofen whilst subjects dosed with STX209 are not. For potential clinical use, our findings suggest that STX209 has the advantage of being a biologically defined and active enantiomer.

## 1. Introduction

**Figure 1:**
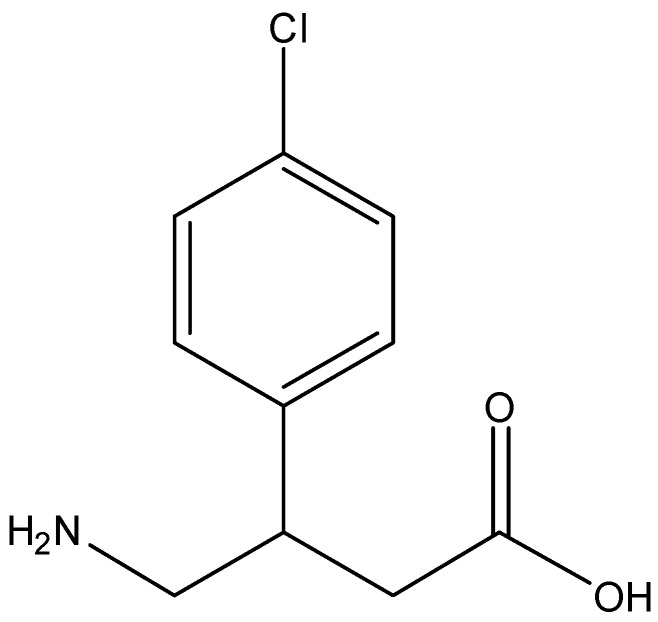
Structure of racemic baclofen.

Baclofen (Figure 1), (±)-4-amino-3-(p-chlorophenyl)-butanoic acid, is a racemic drug, comprising equal amounts of the R- and S-enantiomers, approved over thirty years ago for the treatment of spasticity in adults and children as young as 12 years of age (Lioresal®, NDA 017851, USA FDA 1977). 

STX209 is an exploratory drug comprising the single, active R-enantiomer of baclofen which is being evaluated by Seaside Therapeutics, Inc. in later stage clinical trials for the treatment of fragile x syndrome (FXS) and autism spectrum disorder (ASD) [1].

Information on the clinical pharmacokinetics and metabolism of baclofen has been available for some time [2,3,4,5,6,7,8,9,10]. Information on the clinical disposition of baclofen was first described by Faigle and Keberle in 1972 following a 40 mg single oral dose containing ^14^C-baclofen. The drug showed complete excretion in the urine and feces within three days with the bulk of the dose being excreted in the urine (approximately 80%). Some 85% of the dose was excreted as unchanged drug with one major metabolite identified as the deamination product, 3-(4-chlorophenyl)-4-hydroxybutyric acid [3]. In total, metabolites constituted less than 10% of the material excreted in urine. 

Pharmacokinetic characteristics for baclofen in human volunteers indicate rapid absorption (T_max_ 2 h), dose proportional exposure in the 10–40 mg oral dose range, and rapid elimination (plasma elimination half-life 3.5 h) [4]. Renal clearance is high and equals creatinine clearance indicating the predominance of renal clearance as the chief elimination mechanism of baclofen [2]. Only in more recent times have stereoselective analytical methods been available to measure the enantiomers of baclofen. Pharmacokinetic parameters of the R- and S-baclofen enantiomers after a 20 mg oral dose of baclofen to normal human volunteers indicated similar plasma elimination half-lives (5.3 and 5.1 h respectively) but a slightly higher urinary excretion of R-baclofen relative to S-baclofen [11].

We report interesting new findings from a metabolism and pharmacokinetic investigation in which human volunteers, dosed with either STX209 or baclofen in a cross-over design, show a stereoselective difference in the metabolism and pharmacokinetics of the enantiomers of baclofen. 

## 2. Results and Discussion

Pooled human volunteer plasma and urine samples were analyzed by LC-MS and the collected data were searched against a list of predictable phase I and II metabolites. The list included transformations by oxidative deamination, dechlorination, monohydroxylation, dechlorination-monohydroxylation, and oxidative decarboxylation, and glucuronide, sulfate and glutathione conjugation. For unexpected biotransformations, neutral loss scanning was implemented using 29 and 73 as the neutral losses in negative mode and a loss of 35 in positive mode. The neutral losses of 29 and 73 corresponded to losses of methyleneimine (CH_2_NH) and methyleneimine-carbon dioxide (CH_2_NH-CO_2_); these neutral losses were used to screen for transformations occurring either at the carboxylic group or at the aromatic ring of STX209 or baclofen. The neutral loss of 35, which corresponded to losses of water and ammonia (H_2_O+NH_3_), was used to search for modifications at the aromatic ring, amino and carboxylic groups. The neutral scan data did not show baclofen-related metabolites in human plasma and urine samples based on the expected chlorine isotope pattern in the mass spectral data. 

**Figure 2:**
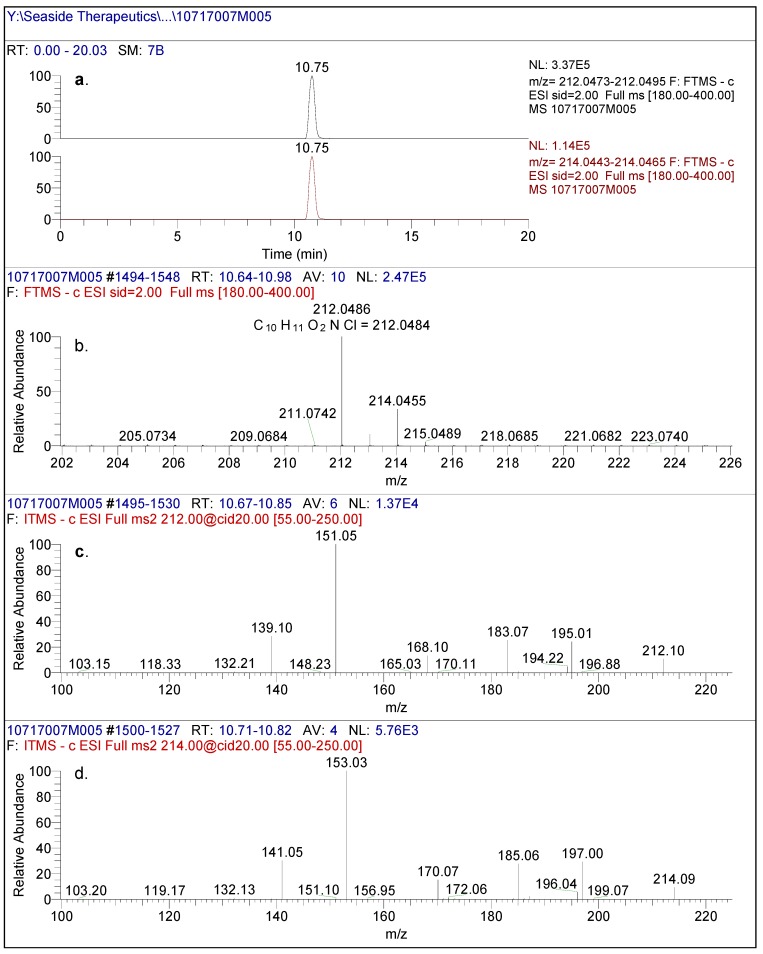
(**a**) High resolution extracted ion chromatogram of baclofen reference standard. (**b**) full scan LC/(-)ESI-FTMS of baclofen reference standard. (**c**) (-)CID-MS/MS spectral data using [M-H]- (212m/z) as the precursor ion and (**d**) (-)CID-MS/MS spectral data using [M-H]- (214m/z) as the precursor ion.

**Figure 3:**
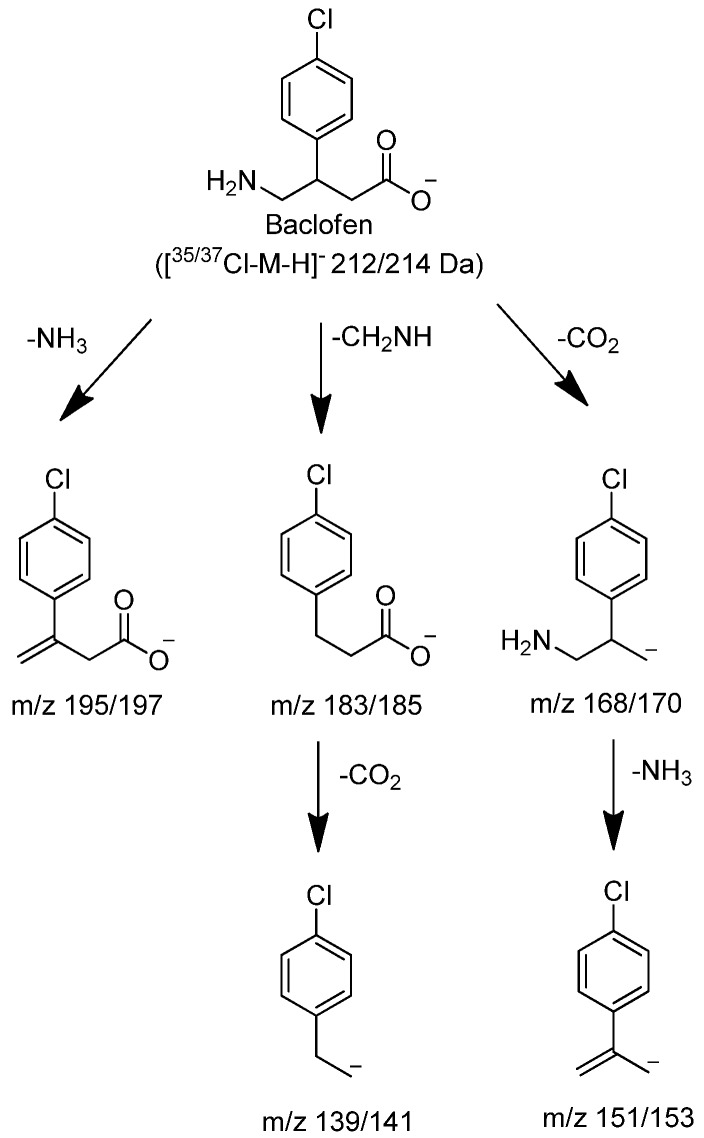
Proposed characteristic (-)CID-MS/MS fragmentations for baclofen.

In negative mode high resolution mass spectrometry, an authentic sample of racemic baclofen produced an accurate mass of the deprotonated molecular ion at m/z 212.0486, consistent with a molecular formula of C_10_H_11_O_2_NCl (0.80 ppm) (Figure 2). The 1/3 ion ratio between m/z 214.0455 and 212.0486 confirmed the presence of chlorine in the molecule. The product ion spectral data of baclofen at m/z 212/214 by CID produced intense ions at m/z 195/197, 183/185, 168/170, 151/153 and 139/141. The most abundant fragment ion was at m/z 151/153. These ions corresponded to neutral losses of carbon dioxide, ammonia and methanimine which were consistent with the structure of baclofen as shown in Figure 3. Similar analysis of an authentic sample of STX209 demonstrated identical full scan and fragmentation mass spectral data (data not shown). 

Of the metabolites predicted *in-silico*, only an oxidative deaminated metabolite (M1) and a glucuronide of M1 (M2) were identified by LC-MS in the human volunteer plasma and urine samples. M1 (3-(4-chlorophenyl)-4-hydroxybutyric acid) was identified as a prominent metabolite in human plasma and urine after administration of racemic baclofen. M1 was not found in human plasma or urine after administration of STX209, indicating enantioselective oxidative deamination. A minor metabolite M2 (a glucuronide of M1) was also found, in trace amounts, in human urine after administration of racemic baclofen. 

### 2.1. M1

**Figure 4:**
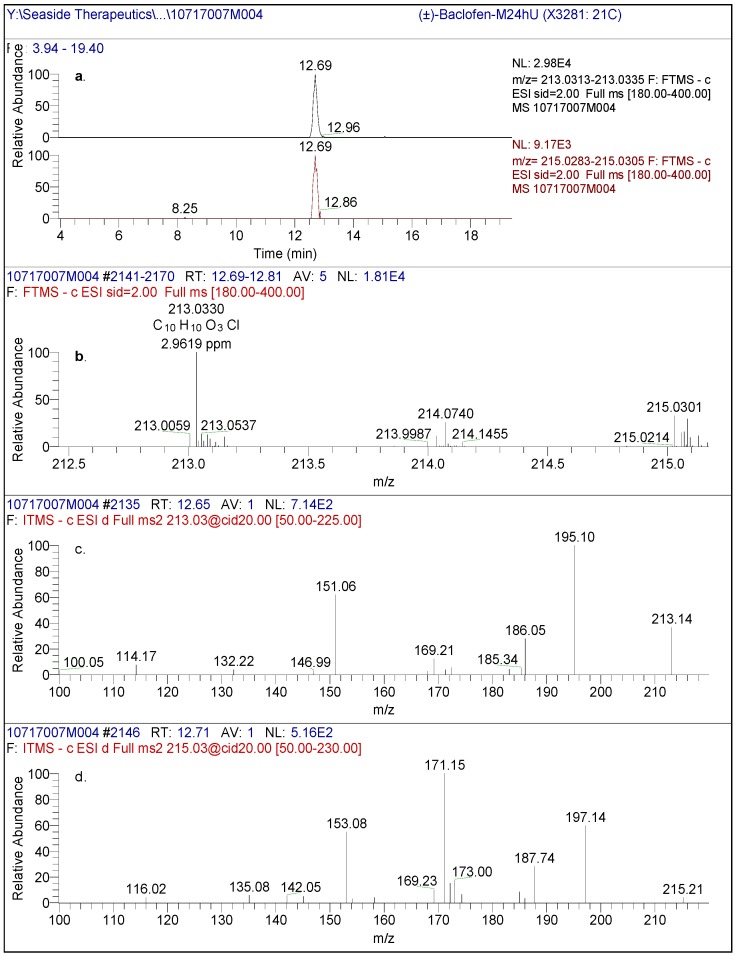
(**a**) High resolution extracted ion chromatogram of pooled human urine (0-24 hr) after administration of racemic baclofen using the exact mass of [M1-H]- and [M1+2-H]- (**b**) full scan spectrum of the chromatographic eluting peak at 12.69 min. (**c**) (-)CID-MS/MS spectral data using [M1-H]- (213m/z) as the precursor ion and (**d**) (-)CID-MS/MS spectral data using [M1-H]- (215m/z) as the precursor ion.

The LC-MS peak eluting at approximately 12.7 min, from a reverse phase HPLC method (Method A), in human plasma and urine from racemic baclofen-treated volunteers was designated as M1 (Figure 4). The LC-MS of M1 produced an accurate mass of the deprotonated molecular ion at m/z 213.0330, consistent with a molecular formula of C_10_H_10_O_3_Cl (2.96 ppm). The molecular weight of M1 is approximately 0.984 Da higher (corresponding to loss of NH_2_ and gain of OH) than that of baclofen, indicative of an oxidative deamination metabolite of baclofen. The product ion spectral data of M1 by CID is depicted in Figure 4. 

**Figure 5:**
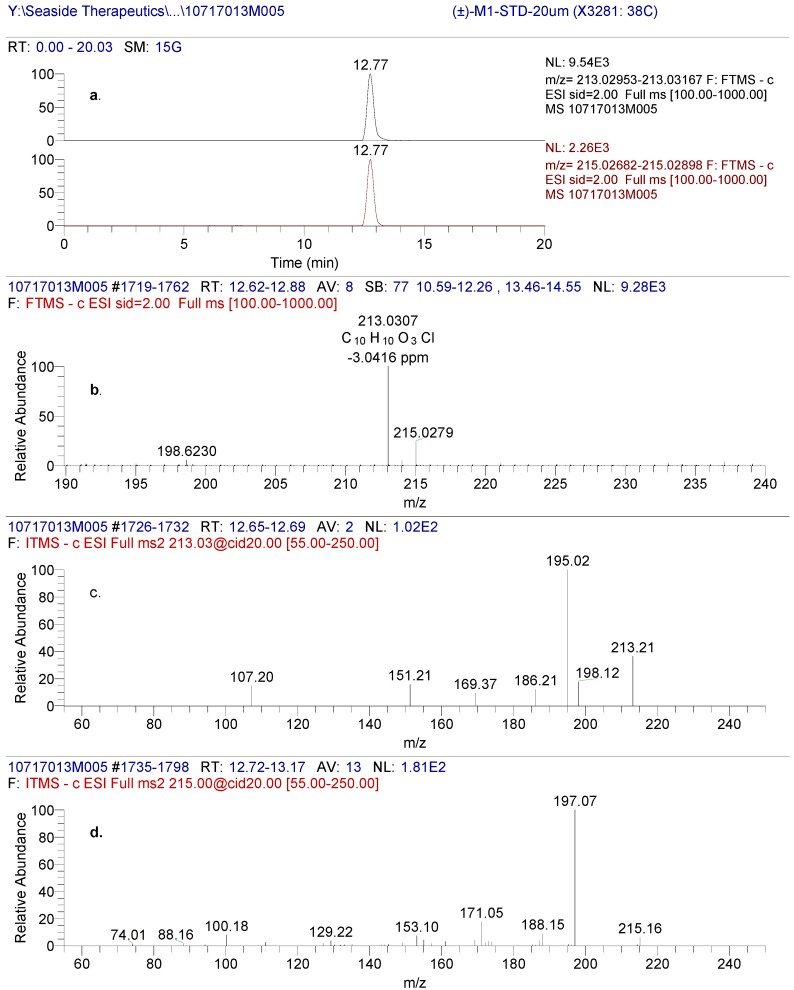
(**a**) High resolution extracted ion chromatogram of (±)-3-(4-chlorophenyl)-4-hydroxybutyric acid reference standard (**b**) Full scan of the precursor ion displaying a characteristic pattern of a compound containing chlorine (**c**) Fragmentation spectrum of ^35^Cl precursor and (**d**) fragmentation spectrum of ^37^Cl precursor ion.

**Figure 6:**
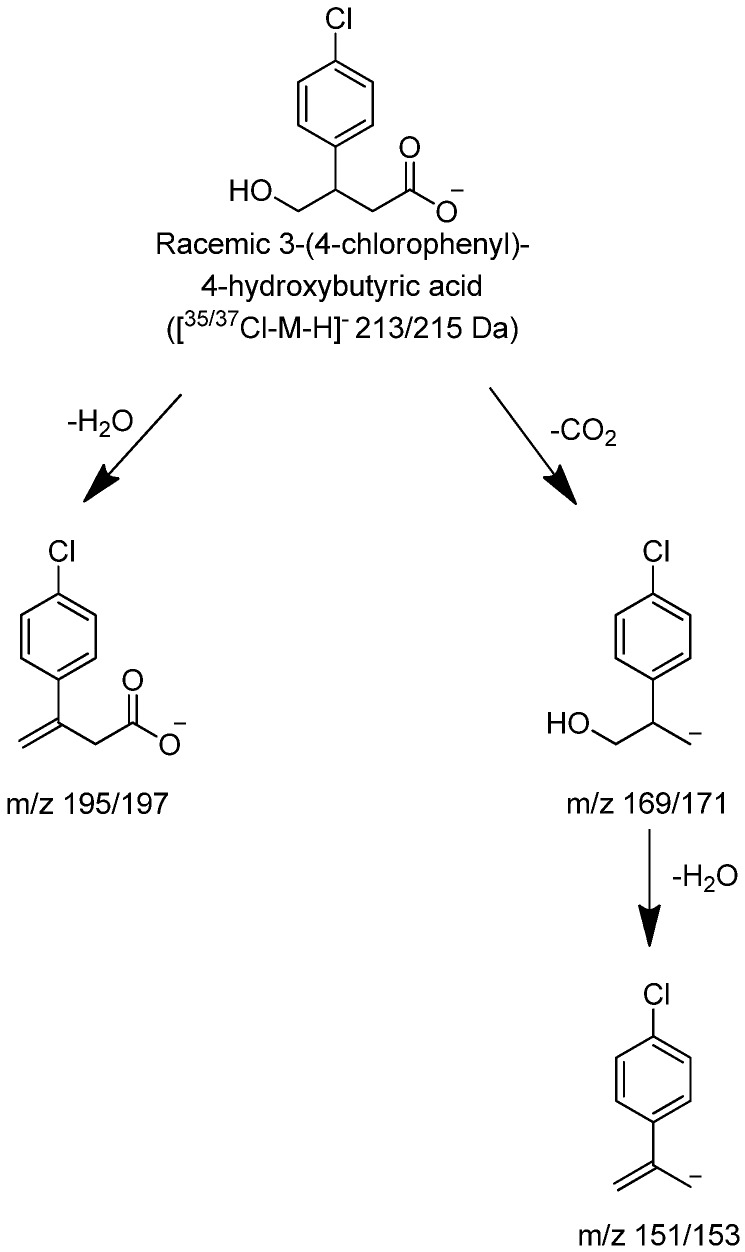
Proposed characteristic (−)CID-MS/MS fragmentations for (±)-3-(4-chlorophenyl)-4-hydroxybutyric acid.

The HPLC retention time, LC-MS full scan and product ion scan data of M1 matched those of 3-(4-chlorophenyl)-4-hydroxybutyric acid reference standard (Figure 5 and later section on the reference standard Figure 6) which further confirmed the structure of the M1 metabolite. Therefore, M1 was identified as 3-(4-chlorophenyl)-4-hydroxybutyric acid but the chirality of the metabolite required further confirmation. 

**Figure 7:**
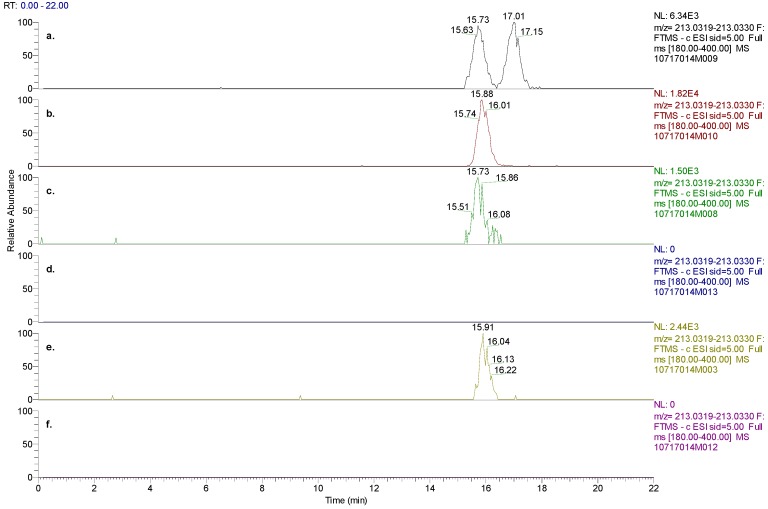
(**a**) Chiral chromatographic separation of M1 enantiomers present in racemic M1 standard, (**b**) S-M1 standard, (**c**) plasma 0–12 hrs pooled samples from human subjects dosed with baclofen, (**d**) plasma 0–12hrs pooled samples from human subjects dosed with STX209, (**e**) urine 0–48 hrs pooled samples from human subjects dosed with baclofen, and (**f**) urine 0–48 hrs pooled samples from human subjects dosed with STX209. The experimental results demonstrated the absence of M1 metabolite in subjects dosed with STX209.

In human plasma and urine samples from volunteers that were administered STX209, the (Method B) HPLC retention time of R-baclofen matched its reference standard and thus the chirality of the molecule was confirmed (data not shown). However, M1 was not detected in the human plasma or urine after administration of STX209 to healthy volunteers (Figure 7).

In order to confirm whether the M1 metabolite was an S- or R-enantiomer, a new chiral HPLC method (Method C) was developed to separate a M1 racemic mixture into its enantiomeric components while avoiding interferences from baclofen. With a new chiral method that employed a Chiralcel OJ-RH column, the racemic M1 standard was separated into S-M1 eluting at 15.7 min and R-M1 eluting at 17.0 min (Figure 7). The HPLC retention time (15.9 min) of S-M1 reference standard matched with S-M1 in the racemic M1 standard. The metabolite M1 discovered in human plasma and urine samples after administration of baclofen separated into one peak with the new chiral method, which matched with the retention time of S-M1 reference standard. In human plasma and urine after administration of STX209, neither S-M1 nor R-M1 was observed. The above findings further confirmed that M1 in human plasma and urine after administration of baclofen is the result of an enantioselective metabolic transformation in which the S-enantiomer of baclofen is transformed by oxidative deamination to S-M1.

### 2.2. M2

In the human urine samples, after administration of racemic baclofen, an LC-MS/MS peak eluted at approximately 8.8 minutes under a non-chiral chromatographic method. It was designated as M2. The negative mode LC-MS of M2 produced an accurate mass of the deprotonated molecular ion at m/z 389.0655, consistent with a molecular formula of C_16_H_18_O_9_Cl (2.72 ppm). The molecular weight of M2 was 176 Da higher than that of M1, indicative of a glucuronide conjugate of M1. The product ion spectral data of M2 by CID showed the characteristic product ion at m/z 213/215, 176 Da less than that of M2, indicating the loss of a glucuronic acid moiety from M2. Product ions at m/z 195/197 and 151/153 from the MS3 experiments of ions m/z 213/215 were the same as those from M1, indicating that M2 is the result of glucuronidation of M1. The glucuronidation position could not be determined solely based on the MS data. M2 was not detected in human plasma after administration of baclofen or in human plasma and urine after administration of STX209.

### 2.3. (±)-3-(4-Chlorophenyl)-4-hydroxybutyric Acid Reference Standard

The negative mode LC-MS of (±)-3-(4-Chlorophenyl)-4-hydroxybutyric acid produced an accurate mass of the deprotonated molecular ion at m/z 213.0307, consistent with a molecular formula of C_10_H_10_O_3_Cl (-3.04 ppm Figure 5). The CID product ion spectra produced significant fragments at 151/153, 169/171 and 195/197. It is hypothesized that the fragment at m/z 195/197 was produced through loss of either H_2_O. The product ions at m/z 151/153 through loss of carbon dioxide and water were the same as those from baclofen through loss of carbon dioxide and ammonia. The product ions at m/z 169/171 from (±)-3-(4-Chlorophenyl)-4-hydroxybutyric acid were 1 Da ( NH_2_, and +OH) higher than product ions at m/z 168/170 from baclofen.

### 2.4. Human Hepatocyte Metabolism

Both STX209 and baclofen were not metabolized in 4 h incubations (98–101% remaining) with viable and metabolically competent human hepatocytes. A similar set of experiments, not included in this article, with pooled human liver microsomes showed the same outcome.

### 2.5. Semi-Quantification of Baclofen, STX209 and S-M1 in Human Plasma and Urine

The presence of baclofen, STX209 and S-M1 were also investigated by LC-MS/MS MRM experiments and their concentration levels estimated in the 0-12-hr pooled plasma samples and the 0-48 hrs pooled urine samples from human volunteers dosed with either baclofen or STX209 (Table 1).

**Table 1:** Concentration of R- and S-baclofen, and S-M1 in human plasma and urine after administration of either racemic baclofen or STX209. Data provided to one decimal place or 3 significant figures. *Urine sample was considered contaminated.

Compound Administered	Subject ID	*R-*baclofen (ng/mL)		*S-*baclofen (ng/mL)		*S-*M1 (ng/mL)	
		Plasma	Urine	Plasma	Urine	Plasma	Urine
Racemic Baclofen 10 mg	P1-01	45.2	1880	35.6	1400	24.9	254
	P1-04	42.4	1030	39.9	806	27.5	146
	P1-06	35.5	1120	31.2	892	19.5	183
	P1-07	36.9	1230	32.1	857	18.7	142
	P2-02	42.7	514	35.9	460	25.1	75.1
	P2-05	40.3	652	34.1	553	16.4	99.4
	P2-08	37.5	552	31.6	457	20.1	95
	Mean	40.1	997	34.3	775	21.7	142
	SD	3.6	482	3.1	333	4.1	61.6
STX209 5mg	P1-02	40.1	1610	-	370*	-	-
	P1-03	37.7	866	-	-	-	-
	P1-05	36.4	1730	-	-	-	-
	P1-08	31.3	892	-	-	-	-
	P2-01	35.1	942	-	-	-	-
	P2-06	35.4	446	-	-	-	-
	P2-07	31.6	422	-	-	-	-
	Mean	35.4	987				
	SD	3.2	513				

"-" signifies the analyte was not detected.

In the per subject pooled plasma samples after administration of racemic baclofen, S-baclofen concentrations were slightly lower than those of R-baclofen (ranges 31.2 to 39.9 *versus* 35.5 to 45.2 ng/mL respectively), whilst S-M1 concentrations ranged from 41% to 88% of the S-baclofen concentrations illustrating the prominence of this metabolite. In the corresponding pooled urine samples, similar trends were evident (S-baclofen and R-baclofen levels 457 to 1400 *versus* 514 to 1880 ng/mL respectively; S-M1 concentrations 5–55% of the S-baclofen concentrations). Cumulative urinary excretion was evaluated for R- and S-enantiomer concentrations after each racemic baclofen and STX209 dosing for up to 48 h. The largest accumulation of R- and S-enantiomer occurred mostly within the first 12 h, indicating similar clearance rates for racemic baclofen and STX209. Though cumulative excretion of each enantiomer was variable between volunteers, approximately 40% of the administered dose of both racemic baclofen and STX209 was eliminated intact. The urinalysis further supports no conversion of R-baclofen to S-baclofen.

In the pooled plasma samples after administration of STX209, R-baclofen concentrations ranged from 31.3 to 40.1 ng/mL and from 422 to 1730 ng/mL in pooled urine samples. No S-baclofen or S-M1 were detected in the plasma and urine samples from the STX209 dosed volunteers with the exception of subject P1-02 in which 370 ng/mL of S-baclofen was detected in the 0-48 hour urine pool, probably due to contamination.

### 2.6. Quantification and Pharmacokinetics of Plasma R- and S-Baclofen

**Figure 8:**
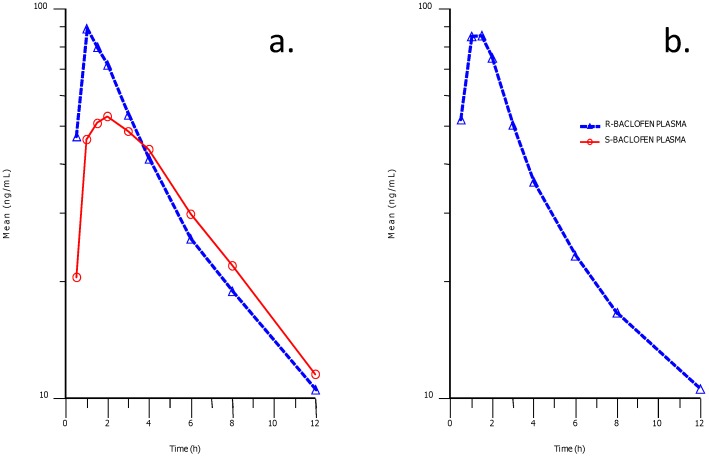
Phamacokinetic profiles in human plasma after an oral dose of baclofen (**a**) and STX209 (**b**). The solid profiles represent the plasma concentration of the S-enantiomer and the dashed lines represent the R-enantiomer.

Stereoselective plasma concentration-time profiles for R- and S-baclofen after racemic baclofen administration indicated a difference in the early part of the profile between the two enantiomers (Figure 8). Higher concentrations and exposures of R-baclofen were attained compared to S-baclofen in the early part of the profiles up to 3 h post-dose.

Thereafter exposures and plasma clearance were almost identical between the two enantiomers. This apparent difference could be explained by an absorption difference between the two enantiomers or alternatively as we believe due to a difference in metabolic clearance to metabolite S-M1 in the case of the S-baclofen. 

These points are further illustrated in the pharmacokinetic parameters estimated (Table 2) in which S-baclofen C_max_ was about 60% that of R-baclofen following racemic baclofen administration, and AUC_last_ was 90%. 

**Table 2:** Pharmacokinetic parameters of human subjects dosed with baclofen or STX209. Data provided to one decimal place or 3 significant figures. Dose administered: 5 mg of STX209 as an oral disintegrating tablet or 10 mg tablet of racemic baclofen in an open label, two treatment, two period, two sequence crossover study.

Compound Administered	Subject ID	Analyte	t_½_ (1/h)	C_max_ (ng/mL)	AUC_last_ (h*ng/mL)
Racemic Baclofen 10 mg	P1-01	R-BACLOFEN PLASMA	5.5	86.8	489
	P2-02	R-BACLOFEN PLASMA	4.7	129	543
	P2-05	R-BACLOFEN PLASMA	5.0	66.6	416
	P1-06	R-BACLOFEN PLASMA	4.5	69.4	328
	P1-07	R-BACLOFEN PLASMA	5.4	103	417
	P2-08	R-BACLOFEN PLASMA	5.1	112	427
		Average	5.0	94.6	437
		Standard Deviation	0.4	22.6	66.8
STX209 5 mg	P2-01	R-BACLOFEN PLASMA	5.6	112	481
	P1-02	R-BACLOFEN PLASMA	6.2	115	495
	P1-05	R-BACLOFEN PLASMA	5.2	100	458
	P2-06	R-BACLOFEN PLASMA	6.3	84.4	341
	P2-07	R-BACLOFEN PLASMA	5.8	85.4	371
	P1-08	R-BACLOFEN PLASMA	5.2	71.8	365
		Average	5.7	94.8	418
		Standard Deviation	0.4	15.6	61.2
Racemic Baclofen 10 mg	P1-01	S-BACLOFEN PLASMA	5.5	56.9	470
	P2-02	S-BACLOFEN PLASMA	4.7	59.5	473
	P2-05	S-BACLOFEN PLASMA	4.8	46.5	372
	P1-06	S-BACLOFEN PLASMA	4.1	59.3	301
	P1-07	S-BACLOFEN PLASMA	4.8	68.9	372
	P2-08	S-BACLOFEN PLASMA	4.9	55.3	372
		Average	4.8	57.8	393
		Standard Deviation	0.4	6.6	60.7

Terminal plasma elimination half-life was the same across both enantiomers and treatments as reported by others in the literature [12]. Stereoselective urinary elimination data taken from the pooled sample analysis over 48 h following baclofen administration indicated that there were no large differences between the two baclofen enantiomers in the major urinary route of elimination (514–1880 *versus* 457–1400 ng/mL of R- *versus* S-baclofen Table 1) but sufficient evidence to suggest that S-enantiomer elimination was lower to account for a proportion being metabolically transformed. Data in the literature (Faigle and Keberle, 1972) obtained after a radiolabeled oral dose of baclofen confirm that urinary elimination is the major route of baclofen and baclofen metabolite clearance.

## 3. Experimental Section

### 3.1. Reagents and Standards

Baclofen-d_4_ (internal standard with four deuterium atoms in the aromatic ring), (±)-3-(4-Chlorophenyl)-4-hydroxybutyric acid sodium salt and S-3-(4-Chlorophenyl)-4-hydroxybutyric acid sodium salt were purchased from Toronto Research Chemicals. S-baclofen was obtained from Sigma-Aldrich. Cryopreserved human hepatocytes were provided by Celsis In Vitro Technologies (Baltimore, MD).

Formic acid, ammonium acetate, and ammonium hydroxide were purchased from J.T. Baker (Phillipsburg, NJ). Acetonitrile was obtained from EM Science (Gibbstown, NJ). Water used in this study was produced on-site at XenoBiotic Laboratories, Inc. through a NANOPure® II (Barnstead Co.) water purification system. Solvents and chemicals were ACS reagent grade or better.

### 3.2. Instrumentation

A Shimadzu LC 10A HPLC system (Shimadzu Corporation, Columbia, MD) coupled to either an LTQ Orbitrap XL Mass Spectrometer (Thermo Fisher Scientific Inc., Waltham, MA) or an ABI Sciex 4000 Q TRAP LC-MS/MS system (Applied Biosystems, Foster City, CA) were used in this study.

### 3.3. Human Hepatocyte Incubations

Cryopreserved human hepatocytes, pooled from three subjects, were thawed prior to use. They were suspended at 1 × 10^6^ viable cells/mL in hepatocyte incubation medium (Celsis In Vitro Technologies) containing the test article (2 and 20 µM STX209 or baclofen) within 24 well cell culture plates (0.5 mL/well). Incubations were conducted in duplicate at 37 ºC in a climate controlled incubator (5% CO_2_/95% air and 95% humidity) for 4 h. Cell viability was checked at the beginning and end of incubation using the trypan blue exclusion method [13].

Incubations were extracted with three volumes of ice-cold acetonitrile, centrifuged at 10,000 g for 10 min and the pellets extracted with 0.5 mL of acetonitrile:water (2:1, v/v). The extracts were combined for LC/MS analysis. Appropriate incubation blanks, negative (no hepatocytes) and positive control (7-ethoxycoumarin and 7-hydroxycoumarin) incubations were performed to verify test article stability and metabolic competence.

### 3.4. Extraction Procedure

Appropriate volumes of R-, S-baclofen, and S-M1 were spiked into blank human plasma samples to create calibration curve standards. An internal standard (baclofen-d_4_) was added to plasma standard curve samples and to the human plasma samples for analysis, and extracted with three volumes of methanol (v/v). The mixture was centrifuged (10,000 g at 4 ºC for 10 min) and the pellets re-extracted with four volumes of methanol:water (3:1, v/v). The combined methanolic extracts were evaporated to dryness under nitrogen, and reconstituted with methanol:water (7:3, v/v) for LC-MS analysis.

### 3.5. Sample Pooling

Two human urine (0–24 h) and two plasma (0–12 h) samples were pooled by dose group across subjects by equal volume for metabolite searching. For semi-quantitation, equal volumes of human urine samples were pooled by subjects across time intervals. All 10 plasma samples in the 0 to 12-h period were pooled according to the trapezoidal AUC pooling scheme as described in the literature by Hop [14].

### 3.6. Metabolite Searching—Method A

A non-chiral method was used to search for predicted metabolites. The data system to acquire data was XCalibur 2.0.7. The method employed an ACE C18 AR column (3.0 µm, 150 × 4.6 mm) with an ACE C18 (3 µm, 3.2 × 10 mm) guard column (Mac-Mod, Chadds Ford, PA). The flow rate used was 0.7 mL/min. To reduce the flow rate for mass spectrometric analysis a post-column ASI 620-P010 (10:1) flow splitter (Analytical Scientific Instruments, El Sobrante, CA) was employed with 1 out of 11 to MS. The column and autosampler temperatures were kept at ambient and at 4 °C respectively. The mobile phase A consisted of 10 mM ammonium acetate in water, with 5% acetonitrile and the mobile phase B consisted of pure acetonitrile. The gradient used was 0% B initial, 0% B at 2 min, 25% B at 25 min and 100% B at 18 min and hold for 0.5 min. The mass spectrometer was a Thermo LTQ Orbitrap XL in negative electrospray ionization mode (ion spray voltage 1.5 kV; capillary temperature 250 °C; capillary and tube lens voltage -40 V & -100 V respectively; sheath gas 10 units; auxiliary/sweep gases were both kept at 5 units).

### 3.7. Chiral Separation of Racemic Baclofen—Method B

A chiral method was used to separate R- and S-baclofen. The instrumentation was the same as the one used for the non-chiral method; but with a Crownpak (CR+) column (5.0 µm, 4.0 × 150 mm) and ACE C18 guard column (3 µm, 3.2 × 10 mm). Post-column ratio 1 out of 2 directed for mass spectrometric detection. The column and autosampler temperatures were set at ambient and 4 °C. Mobile phase A was 10 mM ammonium acetate in water, with 5% acetonitrile and mobile phase B was acetonitrile. The elution gradient (flow rate 0.5 mL/min), 0% B initial condition, 3% B at 2 min, 10% B at 10 min, 15% at 15 min for 5 min (capillary temperature 275 °C; sheath and auxiliary gas flow of 40 and 15 units respectively).

### 3.8. Chiral Separation of Racemic Baclofen Metabolites—Method C

A new chiral method was developed to separate the R- and S-M1 metabolites, if any, from racemic baclofen and plasma interferences. The LC-MS instrumentation was the same as described above. The column used for the separation was a Chiralcel OJ-RH (5.0 µm 2.1 × 150 mm) (Chiral Tech, Daicel Chemical Industries) with a guard column ACE C18 (3 µm, 3.2 × 10 mm). The column and autosampler temperatures were set as in the methods described above. The flow rate of the method was 0.1 mL/min. The mobile phase A consisted of 0.4% formic acid in water:acetonitrile, 80:20 (v/v) and the mobile phase B consisted of 0.1% formic acid in acetonitrile. In order to improve the signal detection in negative mode 0.5% ammonium hydroxide in water:acetonitrile, 80:20 (v/v) at 0.1 mL/min was added as a post-column infusion prior to mass spectrometric detection. The gradient separation was as follows: 3% B as the initial condition, 3% B at 20 min, 100% B at 22 min, 100% at 27 min, 3% B at 29 min followed by 16 min of re-equilibration time. The mass spectrometry parameters were set to the same values as those of the chiral method to separate R- and S-baclofen.

### 3.9. Semi-Quantitation of Metabolites in Human Plasma and Urine

LC-MS/MS MRM analysis was employed for semi-quantitation of R- and S-baclofen, and S-M1 in the 0-12-hr plasma samples pooled by the Hamilton pooling approach and 0-48-hr urine samples pooled by equal percentage. Calibration standard curves were created to quantify the concentrations of R- and S-baclofen (4–125 ng/mL plasma and 125–2000 ng/mL urine), and S-M1 (4–500 ng/mL) with good linearity over the concentration range tested.

### 3.10. Quantitation of R- and S-Baclofen in Human Plasma and Urine

An LC-MS/MS method designed for the chiral analysis of baclofen enantiomers in human EDTA plasma was used. Each baclofen enantiomer was analyzed over a concentration range of 5.0–1000.0 ng/mL using the respective baclofen-d_4_ enantiomers as internal standards.

Human plasma (0.2 mL) was mixed with internal standard and prepared for solid phase extraction by the addition of 0.6 mL of 0.1% formic acid. Samples were then applied to Biotage EVOLUTE ABN 96-well solid phase extraction plates. Loaded plates were washed with 0.1% formic acid and the analytes eluted with 0.8 mL of methanol. The eluant was dried under a stream of nitrogen and reconstituted into 0.1 mL of 1:1 methanol:water (v/v). The reconstituted residue was analyzed using an isocratic mobile phase of 87% 10 mM ammonium acetate and 13% methanol at a flow rate of 600 μL/min through a Daicel Chemical Industries, Crownpak CR(+) column (150 × 4 mm). Both baclofen enantiomers were quantified by monitoring and adding the transitions 214.1 > 151.1 and 214.1 > 179.2. The enantiomers of the internal standard baclofen-d_4_ were monitored using the transition 218.1 > 155.0. Intra-run precision and accuracy were <5%, inter-run accuracy <10%, recovery 45% and 52% for baclofen and baclofen-d_4_ respectively. Baclofen was stable at room temperature and refrigerated (4 °C & −80 °C) for at least 7 days and the processed samples were stable for at least 72 h.

### 3.11. Clinical Study Conduct

Study samples, both plasma and urine, were from 8 healthy human male volunteers (ages 18–40 years) who received an oral dose of 5 mg STX209 as an oral disintegrating tablet or 10 mg tablet of racemic baclofen in an open label, two treatment, two period, two sequence crossover study (Seaside Therapeutics clinical study 209NV103). A single dose of either agent was given under fasting conditions. The two study periods consisted of 4 days each and were separated by 5 days.

The study was conducted according to the U.S. Code of Federal Regulations Guidelines for Good Clinical Practice (Code of Federal Regulations (21 CFR), Parts 50, 54, 56, 312 and 314), the International Conference on Harmonisation (ICH) Guidelines for Good Clinical Practice (ICH Guideline E6), the Declaration of Helsinki on the ethical conduct of medical research and its most recent amendment (Seoul, South Korea, October 2008), and the Belmont Report. 

Prior to study commencement, the protocol and informed consent form were reviewed and approved by Novum Independent Institutional Review Board. All study volunteers provided written informed consent before participating in the study. All safety evaluations throughout the study were found to be within clinically acceptable ranges and no medications were taken during or within one week of study completion. After dosing with racemic baclofen, one adverse event of headache with mild intensity that resolved spontaneously was reported during the study.

## 4. Conclusions

**Figure 9:**
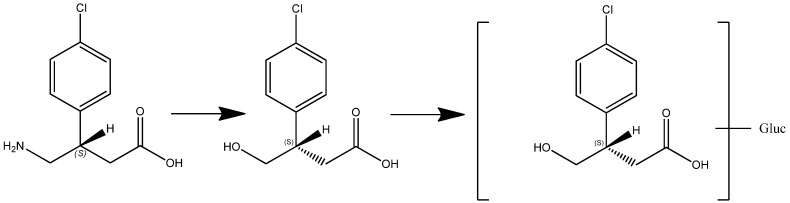
Enantioselective metabolism of racemic baclofen into S-hydroxy baclofen (metabolite M1) and conjugated glucuronide (metabolite M2).

In this article we present new clinical data on the metabolism and pharmacokinetics of the two enantiomers of baclofen. Scientific evidence obtained showed a metabolic difference between baclofen and STX209, the R-enantiomer of baclofen, when dosed orally to human volunteers. Whilst STX209 showed no evidence of metabolic transformation, the S-enantiomer of baclofen when dosed in racemic baclofen underwent metabolism by oxidative deamination to the stereoselective metabolite S-M1 which was detected in volunteer plasma and urine (Figure 7 and Table 1). This metabolite showed levels in plasma that were much higher (41–88% of S-baclofen or 20–44% of racemate) than previously reported for the racemic metabolite of 7% [4]. Small amounts of a hereto unreported glucuronide metabolite were also detected in the urine of volunteers dosed with racemic baclofen. Our results are the first demonstration of stereoselective metabolism of the S-enantiomer of baclofen (Figure 9).

Stereoselective metabolism is a recognized path for metabolic elimination with a number of racemic drugs [15,16,17].

We and others have observed a pharmacokinetic differentiation in the plasma parameters of R-baclofen from S-baclofen [12]. It is our contention that a metabolic difference most likely causes the observed difference in the pharmacokinetic profile of the two enantiomers of baclofen in human volunteer plasma in which the S-enantiomer exhibits a lower C_max_ and lower early plasma exposure compared to the R-enantiomer (Table 2 and Figure 8). This difference probably contributed to the slightly lower urinary excretion of S-baclofen observed. In essence metabolic clearance of S-baclofen to metabolite S-M1 and its glucuronide conjugate by pre-systemic elimination either in the gut wall and/or hepatic first pass could have generated the lower early plasma profile of S-baclofen. Oxidative deamination can be a cytochrome P-450 mediated pathway but equally well could be catalyzed by monoamine oxidase [18,19]. Both enzyme systems have abundance in the gut wall and liver [20,21,22,23,24,25]. In this context, experiments conducted with human hepatocytes and liver microsomes showed no turnover of baclofen. Stereoselective absorption could be a contributory or alternative cause for the pharmacokinetic profile observed. Other workers have noticed a stereoselective difference in the urinary excretion of R-baclofen and proposed stereospecific, active gastrointestinal uptake of R-baclofen as an explanation [11]. The enzymes for oxidative deamination in rats may be different from in humans. Lal and co-workers [26] found that a g-hydroxy metabolite, with modification on the same carbon atom as our metabolite M1, accounted for 1.9 to 3.2% of radioactivity in urine of rats dosed with ^3^H-arbaclofen placarbil, a pro-drug to R-baclofen.

The terminal plasma elimination of the enantiomers of baclofen by renal clearance was similar as would be expected, but the extent of S-baclofen excretion was lower as might be the predicted outcome from the proposed additional stereoselective metabolic component to its clearance.

The biological action of the racemic drug baclofen is known to reside with the active R-enantiomer [27,28,29]. It is exerted by activation of the metabotropic gamma–aminobutyric acid (GABA) B receptor. Our current information shows that patients who receive racemic baclofen will be exposed to additional S-baclofen-derived metabolites which would not be the case with STX209 treatment. For potential clinical use, our findings suggest that STX209 has the advantage of being a biologically defined and active enantiomer. 
